# An exploration of the concept and practice of patient-centered care in medical education in Pakistan

**DOI:** 10.12669/pjms.42.9.13838

**Published:** 2026-09

**Authors:** Afifa Tabassum, Usman Mahboob, Syeda Kauser Ali

**Affiliations:** 1 Afifa Tabassum, MHPE Assistant Professor, Department of Health Professions Education, Liaquat National Hospital and Medical College, Karachi, Pakistan; 2 Usman Mahboob, PhD Professor, Institute of Health Professions Education & Research (IHPER), Khyber Medical University (KMU), Peshawar, Pakistan; 3 Syeda Kauser Ali, PhD Chairperson, Institute of Medical Education, Jinnah Sindh Medical University, Karachi, Pakistan

**Keywords:** Curriculum, Clinical Competence, Education, Hospitals, Medical, Patient centered care, Teaching

## Abstract

**Objectives:**

To explore current understanding of patient centered care (PCC), its teaching and assessment practices and perceived factors influencing PCC learning in undergraduate and postgraduate medical education within a traditional cultural context.

**Methodology:**

An exploratory qualitative descriptive study was conducted from January to September 2024 in public sector hospitals in different provinces of Pakistan. Data was collected through focus group discussions with interns and residents of medicine, surgery and pediatrics selected through purposive sampling. Actor Network Theory informed data analysis and thematic analysis done using Braun and Clarke method.

**Results:**

Six focus groups were held with 33 participants in total. Four main themes and 14 subthemes emerged from analysis: Comprehending Patient-Centered Care, Teaching and Learning Practices, Influencing Factors, and Practice Implications. The central point highlighted was treating the patient as a whole, involving them in their care and giving consideration to personal values and preferences. Some PCC skills identified were technical expertise, communication skills, professionalism and understanding patient’s culture. Current teaching practices were theoretical with limited practical training with insufficient assessment. Curriculum, Faculty, Service, Personal and Patient-related factors were recognized as influencing factors.

**Conclusion:**

This study provides insights into learners’ perspectives and current teaching and assessment of patient-centered care within Pakistani context. The study highlights that to achieve the goal of trained workforce, changes in teaching and assessment practices, curriculum design and policy development at both institutional and national level are required while taking into account the sociocultural context. Addressing these factors could enhance preparation of future healthcare professionals to deliver holistic, patient-centered care.

## INTRODUCTION

The concept of a patient-centered approach to patient care was developed in the 1960s and has since gained extensive recognition.^1^ The Institute of Medicine (IOM) defines patient-centered Care (PCC) as “providing care that is respectful of, and responsive to, individual patient preferences, needs, and values”.^1^ As the definition suggests, this holistic concept of care aims to enhance the quality of healthcare by empowering patients to take an active role in their care. This concept has further evolved into the broader frameworks of person-centered care and family-centered care.^2^

The drive to adopt a patient-centered care model is based on several reported benefits such as open communication, increased compliance, lower health-related costs and improved patient satisfaction.^3^ However its implementation of PCC remains limited at both the individual and institutional levels.^4^ Effective implementation of PCC requires a qualified and trained workforce with a positive attitude. Hence, inclusion of PCC training in medical curricula is being encouraged by international and national accrediting bodies.^5^,^6^ This has led not only to the integration of PCC across different stages of medical education^7^ but also international researches evaluating students’ understanding and perceptions about adequacy and effectiveness of PCC teaching. Although literature is limited, students in these studies acknowledge formal learning methods, role modeling and practical application of theoretical concepts as key factors having an impact on PCC learning. These studies have also identified gaps in curriculum design and implementation for effective PCC training.^8^,^9^

There is limited evidence regarding the status of and perceptions about PCC training in medical education in Pakistan. Available literature reflects predominantly doctor-centered clinical training with an emphasis on the doctor’s leading role in the doctor-patient relationship.^10^ This is further influenced by the distinct cultural and religious perceptions of a physician’s role as a figure of authority in Pakistani society.^11^ Furthermore, studies have reported that the attitude of medical students is more doctor-centered, and there is little change in attitude over the period of five years of medical education.^12^ This lack of evidence of how PCC is currently taught and perceived by learners makes it difficult to develop educational programs or interventions that take into account learner characteristics and the unique cultural context.^13^ Therefore, the objectives of this study were to explore current understanding of PCC, its teaching and assessment practices and perceived factors influencing PCC learning in undergraduate and postgraduate medical education within a traditional cultural context.

## METHODOLOGY

An exploratory qualitative descriptive study was conducted from January to September 2024.

The participants were recruited from medical institutes in various provinces of Pakistan and from major clinical departments (Medicine, Surgery, and Pediatrics) using purposive sampling. Interns who had completed at least three months of house job and residents who had completed at least six months of training were included in the study. Written consent was taken. Focus groups were conducted to include more participants as compared to interviews. Six focus groups were held (three each with interns and residents). One focus group discussion each with residents and interns was conducted in the provinces of Sind, Punjab, and Khyber Pakhtunkhwa (KPK). A semi-structured discussion guide was developed and piloted with five interns. The guide was modified after the pilot and also iteratively adjusted after each session. The focus groups were scheduled at a time that did not coincide with educational activities and clinical work and were moderated by AT and UM, who are trained in qualitative data collection. These were conducted on the hospital premises in a quiet, private room identified by the focal person and in the absence of any faculty or staff. The focus groups lasted between 20 and 40 minutes. There were 33 participants in total, with 16 interns and 17 residents. The researcher established rapport with the participants before initiating the discussion by providing details about the study and obtaining consent. All discussions were audio-recorded for analysis.

### Data analysis:

All discussions were transcribed verbatim. Bilingual discussions were translated into English by one researcher and reviewed by another. Data analysis was done manually using Braun and Clarke’s thematic analysis approach.^14^ Actor–Network Theory (ANT) served as the conceptual framework for data analysis. ANT provided a lens for understanding the human (e.g. students, faculty) and non-human actors (e.g. curriculum, policies, learning environment) and their interactions in shaping PCC education.^15^ Transcripts were coded independently by all authors. Similar codes were then organized into potential sub-themes and themes, which were reviewed and refined through a repetitive consensus-building process. Themes were interpreted and supported with representative participant quotes. Throughout the analysis, the researchers engaged in reflexive discussions to enhance the trustworthiness of the analytical process.

### Rigour:

Lincoln and Guba’s^16^ principles of trustworthiness were adhered to throughout the study. Credibility was established through data source triangulation and researcher triangulation. Dependability for this study was achieved through independent coding and an iterative review of the study findings among all researchers. Confirmability was ensured by the provision of detailed data obtained from the transcript of the focus group session. Transferability was achieved by description of the setting and the context that will help other researchers to transfer findings to their own context. It was also achieved through accurate description of the research results, and verbatim quotes from the participants. It was supported by Purposive sampling ensuring the inclusion of participants who could provide diverse perspectives across different levels of medical education, institutions and provinces of Pakistan.

### Ethics approval:

It was obtained from the institutional review board. (JSMU/IRB/2023/708; April 3, 2023). Written informed consent was obtained from all participants. All the information provided by the participants was kept confidential. Names were omitted in the transcriptions. Quotations in the text were anonymized.

## RESULTS

Six focus groups were held (three each with interns and residents), with 33 participants in total (Table-I). Four main themes and fourteen subthemes emerged from thematic analysis. Interpretation through the lens of Actor–Network Theory (ANT) perspective resulted in the identification of human actors: students, faculty, staff, patients and non-human actors: formal and informal curriculum, learning environment, teaching and assessment practices, institutional policies, and educational resources. These were understood as part of a network that shapes PCC learning. The following is a summary of the combined analysis findings. The four principal themes include Comprehending Patient-Centered Care, Teaching and learning practices, Influencing Factors, and Practice implications (Supplementary Table-II).

**Table-I:** Participant Characteristics

Province	Department	Number of residents	Number of interns
Sindh	Pediatrics	5	5
Punjab	Surgery	6	6
Khyber Pakhtunkhwa	Medicine	6	5
Total		17	16

**Supplementary Table-II:** Themes, sub-themes, categories and quotes

Themes	Subthemes	Categories	Quote
Comprehending Patient-Centered Care	Patient involvement	Patient communication	*The patient should be given full autonomy regarding their decisions and they should be given full disclosure about the treatment. FGD#2* *So I think PCC means that whatever care we need to provide to the patient, we should give them all options and let patient decide according to preference. Second what might be beneficial for patient. FGD#6*
		Patient autonomy	
		Ethical considerations	
	Individualized patient care	Cultural, socioeconomic and religious considerations	*If we think about oncology patients or autoimmune patients, they need individual tailored treatment. So you sort of individualize the patient more in patient centered care. FGD#5*
		Individualized medical needs	
	Totality of care	Continuity of care	*PCC means till the patient becomes ok care should be given even outside the hospital. FGD#3* *Treating the patient as a whole, not only the symptoms, and not only the disease, and have a holistic approach. And we should look at all the aspects. If the patient has been admitted with cough. So we will look not only at cough, but also the development issues. Or any psychological issues that the patient has. FGD#4*
		Holistic care	
		Availability of resources	
	Constituent skills	Technical expertise	*I think empathy first of all. And your knowledge should be good regarding diseases, whether they are rare or whether they are common. FGD#1* *So if we talk about the patient or their attendant. A good approach to managing a patient starts from observation. We observe the patient and pick from there where the child was neglected. Meaning good observation is very necessary. Also timely diagnosis, management, counseling and awareness. FGD#4*
		Communication skills	
		Professionalism	
Teaching and Learning practices	Formal/structured teaching	Theoretical teaching	*We have learnt it theoretically. In undergraduate in the form of lecture. But as a practical we have not learned it in undergraduate. FGD#1*
		Practical skills training	*So although we have a workshop in CPSP (College of Physicians and Surgeons Pakistan) as part of training on communication skills, but that is not tailored according to the patient centered care. FGD#5*
		Assessment	*No, it’s not assessed anywhere. We just have an exam. And it is simply an exam. What can I say? It’s just a formality. FGD#6*
	Informal/unstructured teaching	Role model	*I saw my seniors’ practices with the patients and that’s how we learned it, but there were there was no formal teaching. FGD#2* *Learning is not in any rotation, not like any specific class for this (PCC) or ethics. We are told on bedside that you have to do it this way. FGD#3*
		Situational learning (case dependent)	
	Hidden curriculum	Trial and error	*We are encouraged to do it(PCC), to learn upon it. Practice it, but we don’t actually have the time considering the other things in our course. FGD#4*
		Workload	
		Accountability	
Influencing Factors	Service-related factors	Work constraints	*First of all, there are so many hurdles in reaching the patient itself. That you cannot do it with sincerity. Next is the routine is such that we are so tired. That you think that OK, whatever the patient’s basic requirement is being completed is enough. FGD#6* *And there is no awareness also. Patients don’t know the difference in responsibility of doctor and ward boy. Even if they (patient) can’t get wheelchair it is doctor’s fault. It feels like everything is to be managed by doctor. So we feel like overwhelmed. FGD#3*
		Resource constraints	
	Personal factors	Motivation	*We do not self-evaluate or assess as well…..We don’t take initiative. If we take initiative, then our seniors show interest as well. FGD#6*
		Self-awareness	
	Faculty related factors	Attitude	*Resistance to change. Nobody is ready to change. They are set in their ways. FGD#6*
		Competence	
	Patient related factors	Communication barriers	*There is one thing more that there is a literacy barrier as well. We cannot explain the complex thing into the simple words with the patient. The patient are not that much educated, so most of the people avoid this conversation. FGD#5* *Privacy is most important. If we are taking history from the patient, four relatives are standing in front of them, and if we say please go out and let me talk to the patient, they say no, we know better than the patient. FGD#6*
		Awareness	
		Attitude	
	Curriculum related factors	Structured curriculum	*There is no road map… There is no program director. FGD#6* *Everything is in the course but its proper implementation is not there. Neither in years1-5 or house job. FGD#3*
		Learning opportunities	
Practice Implications	Learning	Curricular change	*It is human nature. That a person. Prepares for the thing on which he is appreciated. For example, if you’re doing the ward work and we know that this professor likes. You know these things, so we then prepare those aspects. So if a program is developed that gives weightage to these things it would be beneficial. FGD#6*
		Suggested teaching/learning strategies	
		Change in assessment	
	Sustainability	Advocacy	*At the end of the day patients should be educated and their attendants also. FGD#2* *Every senior doctor should have a few residents. And this that doctor should be held accountable by the head of department. And there should be a monthly update of what the residents have achieved and what is left? It should be formal. There is no other way. FGD#6*
		Policy	

### Comprehending patient-centered care:

This first key theme “Comprehending Patient-Centered Care” with four subthemes: Patient involvement, Individualized patient care, Totality of care and constituent skills provides deeper insights into participants’ understanding of PCC, including the core knowledge, and skills they perceive as essential component. Participants had varied understanding of the term PCC, but the main emerging point was treating the patient as a whole and not just focusing on the disease. This could be achieved through involving patients in their care and giving consideration to cultural, socioeconomic, religious, and personal values and preferences. One participant comprehensively stated: *“(in PCC) There is basically the involvement of the patient and all steps of the management plan. So that would include consideration being given into the choices, the wishes, and the preferences of the patient. The physical and the emotional needs that the patient might have during the long-term management and the involvement of multidisciplinary teams, and formulating and tailoring an individualized management plan for the individual patient”* (FGD#5). Contextually speaking students of KPK and Punjab were better informed about the term than other areas. Similarly residents had a better idea about the term as opposed to interns. Participants identified what they perceived to be the constituent skills for providing PCC. Both residents and interns emphasized on technical expertise, communication skills, and professionalism. Participants believed that a doctor’s attitude, empathy, and good communication were as important as sound knowledge and expert technical skills. *“Communication skills should be good so that you can communicate in a language which patient can understand.”* (FGD#3). Family’s importance was also highlighted. *“What the patient wants (the treatment), it is not always his wish. Uh, there are several people involved. Including his family.”* (FGD#6). However, opinion was divided as to how much information should be imparted to the patient. As one participant shared, *“However much I try, I cannot make the patient or the attendant understand (disease or treatment). So, I have assessed that the less knowledge that the patient has, it is easier for them to grasp”* (FGD#2).

### Teaching and learning practices:

Under the second theme addressing current teaching and assessment practices of PCC, participants pointed out the lack of formal teaching of PCC, more so at undergraduate level. Formal teaching was more theoretical with little emphasis or opportunities for practical training. The practical training also focused mostly on communication skills in both undergraduate and postgraduate medical education. According to a resident, *“I guess the more focus would be on how to present your beds, maybe how to investigate patients, go towards the diagnosis, management, but nothing about how to communicate with your patient, to address their concerns, or how to manage those concerns”* (FGD#5). Most of the participants mentioned that as these skills were learnt more through informal teaching including situational learning and role models like faculty and senior students. *“It’s on the job informal training. For example, if we have a patient from Afghanistan. We need a translator. We had an assistant professor who knows the language. Then they will translate. The training will be done, uh, right there on the job”* (FGD#6). Participants pointed out that factors like faculty attitude and workload also impacted the learning of PCC skills. This was more evident at postgraduate level. *“Well I think even if you teach it’s difficult to practice due to patient load. I know this is no excuse, patient rights are important, but we are human being”* (FGD#3).

### Influencing factors:

Factors influencing the teaching and learning of PCC were identified, namely Curriculum, Faculty, Service, Personal and Patient-related factors. It is important to note that majority participants from different provinces reported language, literacy and cultural barriers, attendants’ attitude and biases as important influencing factors. The main cultural value highlighted by majority of participants was the decision making power of patients. Participants pointed out that most of the time the decisive power did not lie solely with the patient and at times there was insistence by family not to even inform patients of their condition. *“What the patient wants the treatment. It is not always his wish. There are several people involved, including his family. One is not free in his decision. Secondly whenever we are treating the patient, to be on the safe side we also have to inform the patient’s relatives. What should happen is that the decision should be the patient’s. But here the family is also involved”* (FGD#6). Other key factors identified were patient load, time and resource constraints. These along with a lack of systems for the provision of PCC resulted in a non-conducive environment. The lack of clarity regarding roles and responsibilities within the healthcare team added to this.

Inadequate teaching of PCC was attributed to it not being a required competency, faculty resistance and the lack of competence in this particular area. *“I think the most important thing that a lot of physicians here don’t have is that they don’t stay up to date with the latest research, because things change. One week, even weeks, months, things change. So here, physicians lack the critical review analysis. I would say of recent research in order to know how to better fit it with your patient”* (FGS#2). One group identified the absence of a residency program director as a cause of unstructured teaching.

Level of personal motivation and personal satisfaction had either a positive or negative impact on PCC learning. *“During our educational activities it is enforced that we should be practicing these things but it is our own personal lacking that we don’t have time to practice it on patients.”* (FGD#4). On the other hand, some participants were aware of their shortcomings. Lack of awareness or frustration with faculty attitude led to low prioritization of PCC. Seniors’ support and guidance as an important influence on PCC learning was also highlighted, particularly in the postgraduate phase. *“I’m a junior resident, so my senior resident helps me whenever I have a problem or whenever there are certain things I don’t know about like centers where we can refer the patient to”* (FGD#4). Additionally, participants reported limited external incentives, such as recognition or institutional support both from point of view of learners and teachers to encourage PCC teaching and learning.

### Practice implications:

Participants suggested several changes in curricular design, teaching/learning, and assessment of PCC. Most of them were of the opinion that training should begin in early medical years with a focus on practical training and early patient contact. *“In order for the country or the health environment to significantly change to a patient-centered one, we have to educate the students from the start”* (FGD#5). Proper weightage should be given to PCC competencies both at the undergraduate and postgraduate levels to ensure priority is given to the learning of these competencies. The curriculum should be designed in a manner that there are opportunities for repeated practice of skills in various settings. The discussion also favored role modeling and apprenticeship. One participant explained further, *“Back in the days, you actually used to shadow a doctor. So, you learn a lot of things from them. Yes. You pick up their bad habits as well, when you pick up their good habits. So, I think that actually helps you a lot more having a role model in front of you”* (FGD#2). It was also suggested that there should be regular assessment of these skills at both undergraduate and postgraduate level, and even beyond that maybe as part of recertification.

Several suggestions were made for the sustainability of PCC. These included patient awareness sessions and changes at policy making level so that they are contextually relevant. *“The wrong way of approaching to fill this gap would be adopting the systems that are currently in place in the Western world, because they would not be a suitable match for us. There is a need for the development of local guidelines, local setups, local infrastructure, and local information suitable for us”* (FGD#5). They suggested that changes were required in healthcare infrastructure and policies that support PCC. There should also be accountability for service providers and teachers.

## DISCUSSION

The purpose of this study was to explore current understanding of PCC, its teaching and assessment practices and perceived factors influencing PCC learning at different stages of medical education. Participants viewed prioritization of patients and their values as the core of patient-centered care, with key components of patient involvement and individualized patient care. In other studies, First-year students similarly described PCC as respecting patient perspectives and preferences in decision-making,^17^ while dental students highlighted the importance of cultural values and social well-being in practice.^18^ Another observation in our study was the inconsistent understanding of the term PCC, echoing findings from earlier research where students perceived it as vague or mere jargon.^17^

Our study participants were aware that effective communication with patients and their families would facilitate patient-centered care. This is reflected in other studies. Sri Lankan medical students rated good communication skills highly as essential skills for a doctor^19^ and patients also preferring a patient-centered communication approach.^20^ This importance was also reflected in the curriculum and teaching practices shared in our study, where participants shared that communication skills was a major component addressed in teaching and training. A key fact to note here is that PCC should not be limited to just communication skills.^21^ However, this broader understanding appeared to be lacking in most of our study participants’ responses and was further reflected in the reported limited emphasis on other PCC components within the curricula.

A component strongly highlighted in our study was the influence of culture in the learning and practice of PCC. Participants indicated that family involvement in decision making and patient care was more within our culture, with family or husband taking the dominating role at times. The extent of family involvement varied among different communities. These cultural expectations influenced the practice of PCC, as participants were required to respect and accommodate these norms. This is a trend also noted in other non-Western cultures.^22^ Cultural awareness is an important aspect of PCC as doctors are expected to be sensitive to the cultural beliefs and expectations of patients. These attitudes start developing in medical school, with the hidden and informal curriculum having an effect.^23^ Some participants acknowledged that culture and ethnicity were covered to some extent during clinical training. But it was highlighted more from a biological point of view, discussing the relationship between race and disease rather than as a social determinant of health. Similar findings were documented in other studies as well potentially leading to stereotyping and biases in medical students.^24^ Another aspect reflected in participants opinions was the literacy level of patients. Participants believed that practicing patient-centered care, including shared decision-making and active patient engagement, may be challenging particularly among patients with limited health literacy hat reduces patients’ confidence and ability to participate in their care. Such cultural assumptions may explain why participants perceive challenges in PCC learning and implementing despite acknowledging its importance. Studies from South Asia and Pakistan reflect and support the role of cultural norms and literacy on PCC practice.^25^

Several enablers and obstacles affecting PCC learning emerged in the study. Participants identified personal and faculty attitude as affecting the learning of PCC. They said that personal motivation impacted their learning of patient-centered attitudes. Similar results were noted in other studies^26^,^27^ in which medical students accepted that despite knowing that PCC was beneficial for the patients, their attitude and motivation was missing. Furthermore, they felt that faculty motivation or the lack thereof also had an influence.^27^

Participants unanimously acknowledged the importance of learning PCC and they put forward several suggestions for facilitating learning of PCC. Reforms were suggested at curricular, institutional, and policy levels. Curricular changes called for early training, repeated exposure and regular assessments. Participants also felt that curricular changes should be implemented through accrediting bodies to ensure uniformity. Similar recommendations were given by other health educators who believe it is essential to ensure that medical students are skilled enough to provide care that extends beyond the traditional healthcare model.^28^ Participants of our study believed that the mindset required to implement PCC needs a supportive work environment, incentives, and healthcare systems designed to deliver PCC, an observation that is in agreement with other studies.^29^ Meeting the healthcare system challenges, like time and resource constraints requires changes at the policy level so that there is accountability of institutions as well as teachers.^30^

This study adds to literature by providing a comprehensive exploration of learners’ perceptions of PCC, current approaches to its teaching, and the factors influencing its learning within the Pakistani medical education context.

### Strengths

Main strength was the incorporation of multiple viewpoints across the educational continuum and provinces of Pakistan. The study identifies educational, institutional, and sociocultural influences that shape PCC learning. These findings address an important gap in the literature, as evidence on PCC education from Pakistan remains limited.

### Limitations

It was inclusion of only public sector hospitals in Pakistan; hence findings and implications are context-bound. The use of focus groups to gather data from the students was potentially a limitation as some participants may not have felt comfortable in sharing their thoughts or have modified their responses.

## CONCLUSION

This study provides insights into learners’ perspectives and current teaching and assessment of patient-centered care in medical education within the Pakistani context. The study identifies that PCC learning is sporadic and fragmented. The study also concludes that PCC learning is shaped by a complex interplay of curricular, institutional, and sociocultural factors which should be addressed through targeted educational interventions. The findings highlight the need for a more formal, explicit and longitudinal approach to PCC education. Furthermore, to bring about significant changes in the learning of PCC awareness and commitment regarding PCC from faculty and institutional leadership is needed. Educational policies should be developed that address these factors and prioritize the integration of PCC in medical education. This could enhance the preparation of future healthcare professionals to deliver more holistic, patient-centered care.

### Recommendations:

Further studies should include private institutions and investigate the perspectives of other stakeholders (nurses, faculty, leaders, curriculum designers and patients) to develop a more comprehensive understanding of factors influencing patient-centered care education and practice. Additionally, studies evaluating the effectiveness of specific teaching, learning, and assessment strategies for patient-centered care education within the Pakistani context are needed to inform curriculum design.

### Authors’ Contribution:

**AT and UM:** Literature search**,** Data collection was conducted by.

**AT**: Writing the manuscript, critical review.

**UM and SKA:** Critical Review and final approval.

All authors were involved in Data analysis and interpretation, conception and design of the project and accept responsibility for the integrity of the work.
